# Unveiling the Multifaceted Nature of Spirituality: Assessing the Dimensions of the Spirituality Inventory in the Turkish Context—A Preliminary Adaptation and Validity Reliability Study

**DOI:** 10.1007/s10943-025-02313-7

**Published:** 2025-04-24

**Authors:** Halil Ekşi, Dilek Günel, Ece Yağcı Akgündüz, Emre Gürkan, Fadile Zeynep Çavuş, Yasemin Altıntaş

**Affiliations:** 1 Department of Psychological Counseling and Guidance, Marmara University, Istanbul, Türkiye; 2 Department of Psychological Counseling and Guidance, Istanbul Sabahattin Zaim University, Istanbul, Türkiye; 3 Department of Psychological Counseling and Guidance, Fatih Sultan Mehmet Vakıf University, Istanbul, Türkiye; 4 Department of Educational Sciences, Air NCO Vocational HE School, Turkish National Defence University, İzmir, Türkiye

**Keywords:** Spirituality, Dimensions of spirituality, Scale adaptation, Validity, Reliability

## Abstract

The measurement of spirituality, a concept with significant implications for mental health, requires instruments that can assess its complexity and diversity without cultural limitations. This study adapts the Dimensions of Spirituality Inventory (DSI) into Turkish, examining its validity and reliability. A linguistic equivalence study with 43 participants showed coefficients between .79 and .91. The test–retest correlation coefficients of the subscales ranged from .35 to .78. The 53-item scale was then administered to 671 adults, and confirmatory factor analysis indicated an acceptable fit. The internal consistency reliability coefficient was .90. Positive correlations with related scales confirmed acceptable criterion-related validity. The study concluded that the Turkish version of the DSI is a valid and reliable tool for use in scientific research in Türkiye.

## Introduction

Historically, topics such as religion and spirituality have received less attention within the scientific discourse, primarily due to the prevailing positivist paradigms. Nevertheless, since the onset of the twenty-first century, the significance of religion and spirituality in human functionality has gained recognition, catalyzing broader acceptance within scientific circles (Emmons & Paloutzian, 2003; Hill et al., 2000; Miller & Thoresen, 2003; Zinnbauer et al., 1999). Powers (2005) argued that this shift is largely due to therapists recognizing the spiritual dimensions of their clients’ mental health challenges, thereby integrating these insights into clinical practice. In essence, real-world observations have increasingly informed and enriched the scientific study of these areas.

With respect to the emergence of the concept of spirituality, traditional research has centered on the concepts of religion and religiosity. Religiosity refers to practices and experiences linked to transcendent, divine and religious institutions. On the other hand, spirituality was used to describe spiritual practices such as mysticism or nonreligious practices such as séances before it was treated separately in this field (Pargament, 2023). Closer to the present day, different definitions of spirituality have emerged. For example, Benner (1989, p. 20) defines spirituality as “the human response to God’s call for relationship”; Elkins et al. (1988) defines it as “a way of being and experiencing that is characterized by an awareness of the transcendent dimension and identifiable values about self, others and nature”; Hart (1994, p. 23), “the fundamental orientation of an individual’s life, its relation to the ultimate foundations of existence, the transcendence of the human spirit”; Shafranske and Gorsuch (1984, p. 231), “a transcendent dimension in human experience, emerging at moments when the individual seeks the meaning of his or her existence and tries to place the self in a more inclusive ontological context”; Vaughan (1991, p.105), “the subjective experience of the sacred, in contrast to the institutionalization of religion”. Despite the multitude of definitions that exist regarding spirituality, it is a fundamental truth that spirituality has consistently been concerned with the “inner world” and “inner experience” of humanity. It provides a framework for understanding the significance and purpose of a transcendent connection that extends beyond the confines of one’s life and human experience (Sargeant & Yoxall, 2023). This diversity in the definition of spirituality also leads to difficulties in its measurement.

One of the challenges in quantifying spirituality lies in determining whether it should be conceptualized as a singular construct or a multidimensional phenomenon. Research indicates that spirituality is not perceived as a universal truth (Ammerman, 2013; Eisenmann et al., 2016; Steensland et al., 2018; Zinnbauer et al., 1997). For instance, Zinnbauer et al. (1997) identified thirteen distinct categories of spirituality based on their studies across various religious and professional groups. Similarly, Eisenmann et al. (2016) proposed a hierarchical model of spirituality with forty-four dimensions initially, which were refined to ten and three dimensions in subsequent analyses. Ammerman (2013) supports these findings, suggesting that spirituality comprises multiple dimensions, which was echoed by Steensland et al. (2018), who described seven unique forms of spirituality. Collectively, these studies highlight the complex and rich nature of spirituality.

Although often associated with religion, spirituality encompasses broader experiences such as the pursuit of meaning, transcendent experiences, commitment, respect, and gratitude toward a mysterious entity, and personal transformation. For example, Pargament (1997) defines spirituality as the quest for the sacred, which is crucial both within and outside religious contexts. Unlike religion, which may involve various sacred and secular pursuits, spirituality strictly addresses the sacred, aiding individuals in finding deeper meaning in life and death through personal and interpersonal connections. Furthermore, spirituality and religion are considered vital in managing life challenges and understanding their causes, influencing coping mechanisms and responses to life events (Pargament, 1997). Research on the impact of spirituality on mental health, such as that of Larson and Larson’s (2003) analysis of more than 1200 studies, suggests that spirituality fosters inner peace and aids in managing stress and anger, thereby offering protection against depression. More recent studies, including that of Coppola et al. (2021), revealed positive correlations between spirituality and mental health during the COVID-19 pandemic. The research conducted by Shattuk and Muehlenbein (2020) demonstrates the correlation between spirituality and not only mental health but also physical health.

To accurately measure spirituality and related constructs, numerous scales have been developed globally, such as the Spirituality Expressions Inventory (Macdonald, 2000), the Assessment of Spirituality and Religious Emotions Scale (ASPIRES) (Piedmont, 2004; Piedmont et al., 2008), and the Daily Spiritual Experiences Scale (Underwood & Teresi, 2002), among others. In Turkey, similar efforts have resulted in instruments such as the Spirituality Scale (Şirin, 2018), the Intrinsic Spirituality Scale (Ekşi et al., 2018), and the Maternal Spiritual Coping Scale (Yaman et al., 2018). Recently, the Dimensions of Spirituality Inventory (Wildman et al., 2024), which expands traditional definitions by incorporating diverse religious and spiritual traditions while avoiding culturally biased assumptions, was introduced. This tool aims to universally assess varied spiritual practices and is being adapted for Turkish cultural contexts to enhance the understanding of spirituality locally. Wildman et al. (2024) summarized the diverse perspectives and confusion within the field with his Dimensions of Spirituality Inventory, introducing a measurement tool with cross-cultural validity to the literature. According to the “Perception of Religiosity in Turkey” report by the Ankara Institute and the Istanbul Policy Center, 92.3% of Turkish people identify as Muslim, with 86% expressing unwavering belief in the existence of God. Given Turkey’s diverse cultural landscape and high level of religiosity, it is essential to adapt a comprehensive inventory to measure spirituality in this context.

## Methods

### Study Group

The scale adaptation study encompassed multiple phases and involved participants across four distinct demographic groups. Data for the research were gathered both online and through direct interpersonal interactions. The entire process of scale adaptation was predicated on volunteer participation, and informed consent was duly obtained from all contributors. In the phase dedicated to linguistic equivalence, data were sourced from 43 university students proficient in both English and Turkish. The construct validity phase involved a diverse sample of 671 adults, predominantly female (*83.6%, n* = *561*) and a smaller male contingent (*16.4%, n* = *110*). The sample included 69 individuals to assess criterion validity, with a distribution of 62 females and 7 males. The test–retest reliability study was conducted with 49 participants using convenience sampling. This method is noted for its practicality and cost-effectiveness in recruiting volunteers (Monette et al., 1990). Data collection was primarily conducted via Google Forms, an approach supported by research from Gosling et al. (2004), confirming that online data are generally as reliable and valid as those gathered through traditional methods. Demographic information about the participants in each group is given in Table 1.

**Table 1:** Demographic information of the participants

Group	Gender	Age	N	Total
Group 1	Female	19–70	561	671
CFA	Male		110	
Group 2	Female	21–34	35	43
Linguistic validity	Male		7	
Group 3	Female	19–67	62	69
Criterion validity	Male		7	
Group 4	Female	20–55	48	49
Reliability	Male		1	

### Process

This study focuses on adapting the Dimensions of Spirituality Inventory to the Turkish version and evaluating its psychometric properties. Initially, permission was secured from the original scale developers, followed by ethical approval from the Marmara University Ethics Committee. All the data collected from the participants provided informed consent. The scale was adapted to Turkish by considering the steps recommended to be followed in the translation process of a spiritual scale (Koenig & Al Zaben, 2021). After the translation process was completed, psychometric validity studies of the scale were carried out. All the statistical analyses were not item-based but dimension-based, as in the original study. Thus, all the items were included in the analysis in five main dimensions.

#### Translation Process

During the translation process, weekly meetings were held between the researchers. The first phase involved linguistic equivalence testing, in which the original scale was translated into Turkish by 30 bilingual individuals. The translations were compared, and any differences were reconciled through discussion among the researchers. The reconciled version was back translated into English to ensure consistency. The Turkish version was generated by comparing the back translation, original version, and Turkish version.

A two-step pilot study was conducted to examine the comprehensibility of the scale. The Turkish form was first reviewed by 11 adults, and their opinions on the comprehensibility of the scale items were reviewed. Adjustments were made to the form within the framework of their opinions. Subsequently, the comprehensibility of the scale was re-evaluated item by item by 14 different adults. Within the scope of the comments, definitions of some terms and how they can be interpreted by people with different beliefs were added to the explanation section of the scale. The Turkish version was proofread by a linguist who is fluent in Turkish and Turkish culture. Thus, the translation of the form into Turkish was completed. To assess linguistic equivalence, the finalized Turkish and the original English versions of the scale were administered to 43 bilingual participants at two-week intervals. The results of the linguistic equivalence analysis showed consistency between the English and Turkish forms and are described in the results section.

#### Psychometric Validation Process

For construct validity, data were collected from 671 participants and analyzed using confirmatory factor analysis (CFA) to establish the factor structure’s alignment with the original scale. The scale’s reliability was tested by administering it to 49 individuals twice, two weeks apart, and calculating test–retest reliability coefficients. Cronbach’s alpha coefficient of the scale was also calculated. Criterion validity was evaluated through correlations among the Spirituality Scale (Şirin, 2018), the Spiritual Transcendence Scale (İme et al., 2019), the Mystical Experience Scale (Ünal, 2000), and the Intrinsic Spirituality Scale (Ekşi et al., 2018). All validity analyses are described in the results section.

### Measures

**Dimensions of Spirituality Inventory.** The Spirituality Inventory is the scale adapted for this study. It is a 53-item quantitative spirituality assessment with a 5-point Likert scale. It consists of 5 main dimensions and 21 subdimensions with 3 control items. Main dimensions and subdimensions: Value Ideals, Appreciating Beauty, Axiological Sensitivity, Ethical Growth, Kinesthetic/Kinaesthetic, Truth-Seeking. Belief and Belonging: Belief, Ritual, Religious Tradition. Mysticism: Awe, Mystery, Oneness-Unity, Oneness-Transcendence. Personal: Connection, Meaning, Nonconnectedness, Practices, Worship, Self-Discovery, Self-Transformation. Transcendent Beings; Dead Active, Divine Beings, Spiritual Beings. In the original study, the Cronbach’s alpha reliability coefficient for the whole inventory was found to be *r* = 0.92 (Wildman et al., 2024).

**Spirituality Scale.** The scale was developed for youth and adults to assess various dimensions of human spirituality, such as searching for the meaning of life, connection with the divine (transcendent), searching for the ultimate truth or the highest value, comprehending the mystery of creation, feeling of attachment to God, customization of belief, feeling of unity with the universe, and having 27 items and a 5-point Likert type. The scale consists of a total of seven factors, and the fit values obtained as a result of confirmatory factor analysis are appropriate. The reliability of the scale was as follows: Cronbach’s alpha, 0.90; Guttman, 0.91; Spearman-Brown, 0.89; and test–retest reliability coefficient, *r* = 0.95 (Şirin, 2018). In the current study, the Cronbach’s alpha values for the scale were 0.84 for the total scale, 0.88 for connection, 0.80 for spiritual life, 0.47 for harmony with nature, 0.41 for spiritual fulfillment, 0.56 for searching for the meaning of life, 0.71 for transcendence, and 0.63 for spiritual coping.

**Spiritual Transcendence Scale.** The Spiritual Transcendence Scale developed by Piedmont (1999) and adapted by Ime (2019), which aims to determine individuals’ spiritual transcendence level, consists of 24 items and is scored on a 5-point Likert scale. Universality, connectedness, and worship satisfaction represent the subdimensions of the scale. The internal consistency reliability (Cronbach’s alpha coefficient) is 0.90 for the overall scale, 0.84 for the subdimensions of worship satisfaction, 0.91 for universality, and 0.93 for connectedness. In this study, the Cronbach’s alpha value of the scale was found to be 0.81 for the overall scale, 0.61 for satisfaction with worship, 0.81 for universality, and 0.45 for connectedness subdimensions (İme et al., 2019).

**The Mysticism Scale.** The scale developed by Hood (1975) and adapted by Ünal (2000), which aims to measure individuals’ mystical experience levels, consists of 32 items in a 5-point Likert format, 16 of which are positive and 16 of which are negative. A high score on the mystical experience scale indicates a high level of mystical experience (Ünal, 2000). In this study, Cronbach’s alpha was found to be 0.93 for the overall scale.

**Intrinsic Spirituality Scale.** The intrinsic spirituality scale was developed by Hodge (2003) and adapted by Ekşi et al. (2018). Inner spirituality refers to one’s relationship with God and ultimate transcendence. It is a 10-point Likert-type scale with a total of 6 items with a single factor. There are no reverse-scored items on the scale. A higher total score obtained from the scale indicates that the individual’s inner spirituality is high. Confirmatory factor analyses revealed that the fit indices were acceptable. Cronbach’s alpha internal consistency coefficient was calculated to test reliability. According to the reliability analyses, the Cronbach’s alpha coefficient for the whole scale was 0.95. (Ekşi et al., 2018). In this study, the Cronbach’s alpha value was calculated as 0.94.

## Results

### Linguistic Validity

During the adaptation of the Dimensions of Spirituality Inventory into Turkish, initial studies focusing on linguistic validity were conducted. Both the Turkish and English versions of the inventory were administered to a bilingual cohort of 43 individuals who were proficient in both languages, with a two-week interval between sessions. The analysis of linguistic validity was performed using correlation values and paired *t*-tests across the two administrations, and the results are detailed in Table 2.

**Table 2:** Linguistic validity of the dimensions of spirituality inventory

Inventory	Application	X̄	SD	t	sd	p	*r*
Value	English form Turkish form	48.41 48.95	8.69 7.98	− .851	42	.399	.88*
Belief	English form Turkish form	20.41 20.51	5.43 4.94	− .189	42	.851	.81*
Mystical	English form Turkish form	35.60 35.11	6.44 5.56	.801	42	.427	.79*
Personal	English form Turkish form	49.58 50.11	8.73 7.78	− .887	42	.380	.89*
Transcendent	English form Turkish form	20.18 20.44	5.07 4.96	− .772	42	.444	.90*
DSI Total	English form Turkish form	174.20 175.13	28.20 24.52	− .539	42	.593	.91*

*p < .001

In the realm of linguistic validity, comparative analysis using the *t* test revealed no statistically significant disparity in the mean scores between the English and Turkish versions of the inventory (*t* = 42; *p* < 0.001). Furthermore, a robust positive correlation was observed between these versions, as indicated by a Pearson correlation coefficient of 0.91 (*p* < *0.0*01). Moreover, the analysis extended to the subdimensions of ‘Value’, ‘Belief’, ‘Mystical’, ‘Personal’, and ‘Transcendent’ within the Turkish version of the DSI compared against scores from the original English format. The results showed no significant differences in the mean scores. Consistently, the Pearson correlation coefficients for these subdimensions confirmed significant and substantial relationships across the board. According to the findings obtained, the scale yields similar results in both languages and has linguistic equivalence.

### Construct Validity

**Fig. 1:**
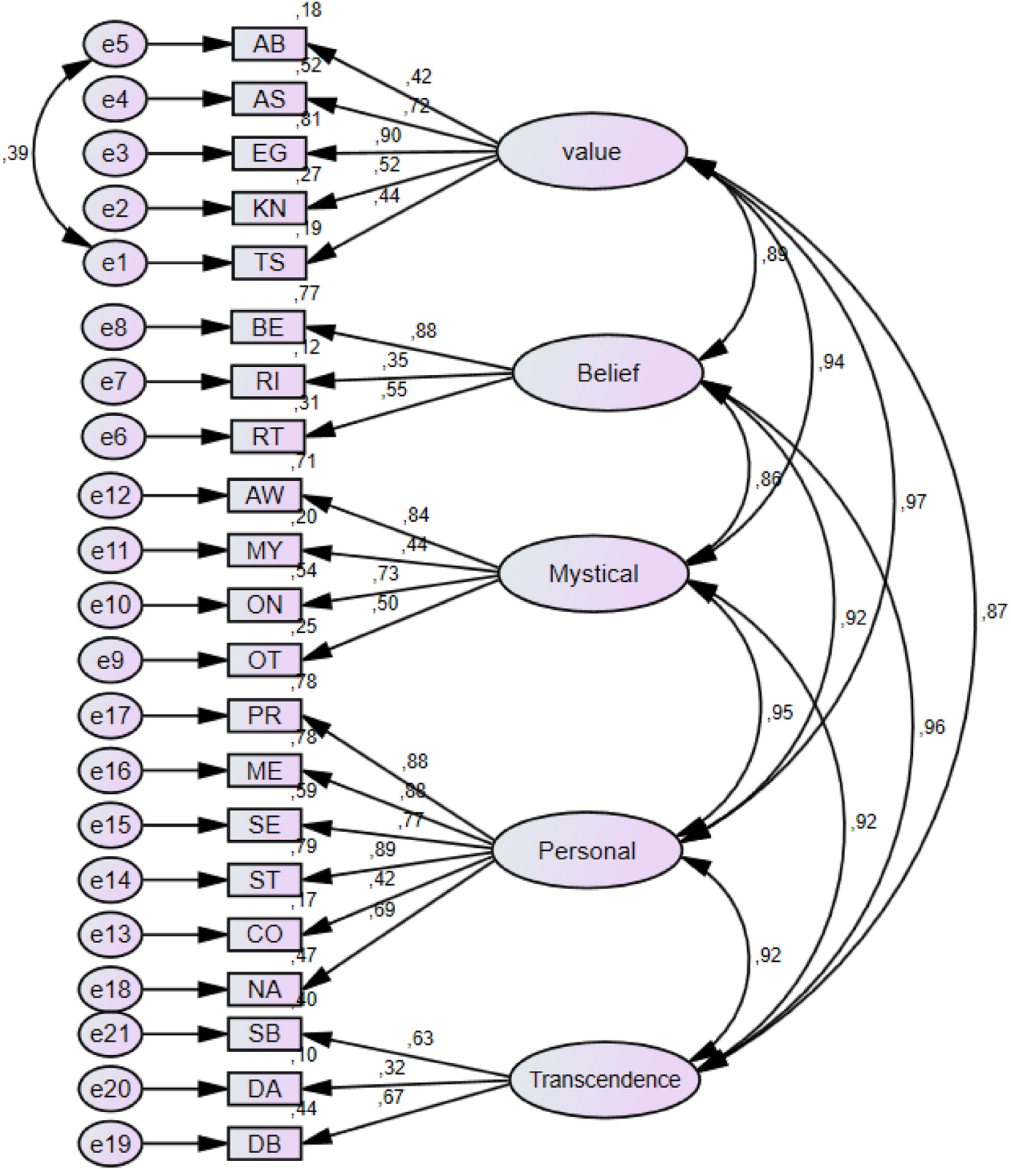
Confirmatory factor analysis model and standardized item factor loads of the DSI

CFA was conducted to measure the construct validity of the Turkish version of the inventory. The fit values obtained after the analysis revealed which is given in the Table 3 that the Turkish version of the inventory had acceptable fit indices (Hu & Bentler, 1999; Tabachnick & Fidell, 2001, 2013). The five-dimensional structure of the inventory in the Turkish sample and the factor loadings of the items are given in Fig. 1.

**Table 3:** The fit values obtained after the CFA

Fit indices	Value
χ^2^/sd	4.29
RMSEA	.070
GFI	.88
AGFI	.85
CFI	.92
TLI	.91

RMSEA, root mean square error of approximation, GFI, goodness-of-fit index, AGFI, adjusted goodness-of-fit index, CFI, comparative fit index, TLI, tucker-lewis index

As illustrated in Fig. 1, the factor loadings for the inventory items range from 0.42 to 0.90, indicating a substantial alignment with the respective factors. Specifically, within the ‘Values’ subdimension (F1), loadings vary between 0.42 and 0.90. For the ‘Belief’ subdimension (F2), the range is 0.50 to 0.85; for the ‘Mystical’ subdimension (F3), it is 0.44 to 0.80; for the ‘Personal’ subdimension (F4), 0.76 to 0.89; and for the ‘Transcendence’ subdimension (F5), 0.60 to 0.65. These factor loadings robustly reflect the extent to which items correlate with their respective underlying constructs.

The obtained factor loadings and CFA fit indices comply with the criteria indicating that the model provides an excellent or acceptable fit. According to the fit index evaluation criteria in the literature (Kline, 2005; Schumacker & Lomax, 2010; Sümer, 2000; Schermelleh-Engel et al., 2003; Hu & Bentler, 1999; Byrne, 2010), the Turkish version of the DSI is psychometrically reliable and valid.

### Criterion Validity

To examine the criterion-related validity of the Dimensions of Spirituality Inventory, two subdimensions of the Mysticism Scale (MES2 and MES5), which are the “informational religious quality factor that leaves a positive impression” and the “sense of divine perfection factor”, two subdimensions of the Spirituality Scale, namely, “Spiritual Coping” and “Search for Meaning” (SSC and SSM); two subdimensions of the Spiritual Transcendence Scale, namely, “Universality” and “Satisfaction with Worship” (STSU and STSS); and the Intrinsic Spirituality Scale (ISS), were utilized. The correlation values between the scores obtained from the scales are presented in Table 4.

**Table 4:** Correlation analysis results between scales

	M	SD	MES2	MES5	SSC	SSM	ISS	STSU	STSS
M			17.91	7.27	21.13	18.53	45.97	34.37	33.20
SD			4.72	1.51	2.43	1.74	16.80	3.93	4.00
Belief and belonging	22.73	3.21	.352**	.308**	.617**	.463**	.136	.509**	.507**
Value ideals	52.76	6.68	.497**	.453**	.594**	.328**	.272*	.569**	.503**
Mystical	39.69	4.96	.268*	.291*	.705**	.406**	.266*	.466**	.373**
Personal	56.76	6.61	.415**	.422**	.809**	.594**	.304*	.611**	.619**
Transcendent beings	19.49	6.62	.397**	.468**	.466**	.475**	.136	.375**	.306*

MES2, Factor of informational religious quality leaving a positive impact, MES5, Sense of divine perfection factor, SSC, Spiritual Coping, SSM, The Search for Meaning, ISS, Intrinsic Spirituality Scale, STSU, Universality, STSS, Satisfaction with Worship

*p < .05, **p < *.01*

Table 4 shows that the “Belief and Belonging” dimension of the Spirituality Dimensions Inventory’s “Belief and Belonging” dimension is positively correlated with the subdimensions of the Mysticism Scale, namely, “the factor of informational religious quality that leaves a positive impression” and (*r* = 0.35; *p* < 0.01), the factor of sense of divine perfection” (*r* = 0.308; *p* < 0.01), “Spiritual Coping” (*r* = 0.61; *p* < 0.01) and “Search for Meaning” (*r* = 0.46; *p* < 0.01), which are the subdimensions of the Spirituality Scale; “Universality” (*r* = 0.50; *p* < 0.01) and “Satisfaction with Worship” (*r* = 0.50; *p* < 0.01) of the Spiritual Transcendence Scale.

The “Value Ideals” dimension is related to the subdimensions of the Mysticism Scale, namely, “the factor of informational religious quality that leaves a positive impression” (*r* = 0.49; *p* < 0.01) and “the factor of sense of divine perfection” (*r* = 0.453; *p* < 0.01); the subdimensions of the Spirituality Scale, namely, “Spiritual Coping” (*r* = . 59; *p* < 0.01) and “Search for Meaning” (*r* = 0.32; *p* < 0.01); “Universality” (*r* = 0.56; *p* < 0.01) and “Satisfaction with Worship” (*r* = 0.50; *p* < 0.01) of the Spiritual Transcendence Scale; and the overall Inner Spirituality Scale *(r* = 0.27*; p* < 0.05*).*

The “mystical” dimension is related to the subdimensions of the Mysticism Scale, namely, “the factor of informational religious quality that leaves a positive impression” (*r* = 0.26; *p* < 0.05) and “the factor of the sense of divine perfection” (*r* = 0.29; *p* < 0.05); the subdimensions of the Spirituality Scale, namely, “spiritual coping” (*r* = . 70; *p* < 0.01) and “Search for Meaning” (*r* = 0.40; *p* < 0.01); “Universality” (*r* = 0.46; *p* < 0.01) and “Satisfaction with Worship” (*r* = 0.37; *p* < 0.01) of the Spiritual Transcendence Scale; and the overall Inner Spirituality Scale (*r* = 0.26; *p* < 0.05).

The “Personal” dimension is related to the subdimensions of the Mysticism Scale, namely, “the factor of informational religious quality that leaves a positive impact” (*r* = 0.41; *p* < 0.01) and “the factor of the sense of divine perfection” (*r* = 0.42; *p* < 0.01); the subdimensions of the Spirituality Scale, namely, “Spiritual Coping” (*r* = . 80; *p* < 0.01) and “Search for Meaning” (*r* = 0.59; *p* < 0.01); “Universality” (*r* = 0.61; *p* < 0.01) and “Satisfaction with Worship” (*r* = 0.61; *p* < 0.01) of the Spiritual Transcendence Scale; and the overall Inner Spirituality Scale (*r* = 0.30; *p* < 0.05).

The “Transcendent Beings” dimension, on the other hand, has a positive effect on the subdimensions of the Mysticism Scale, namely, the “informational religious quality factor that leaves a positive impression” (*r* = 0.39; *p* < 0.01) and the “sense of divine perfection factor” (*r* = 0.46; *p* < 0.01); the subdimensions of the Spirituality Scale, namely, “Spiritual Coping” (*r* = . 46; *p* < 0.01), “Search for Meaning” (*r* = 0.47; *p* < 0.01), “Universality” (*r* = 0.37; *p* < 0.01) and “Satisfaction with Worship” (*r* = 0.30; *p* < 0.05) of the Spiritual Transcendence Scale.

### Reliability

Test–retest reliability analysis was conducted in the scope of reliability studies. To evaluate the consistency of the Turkish version of the DSI over time, the Turkish version of the DSI was administered to a group of 49 participants at two-week intervals. The mean scores, standard deviations and *t*-test results obtained between the first and second administrations revealed consistently high levels of reliability, with Pearson correlation coefficients ranging from 0.35 to 0.78 for the various dimensions of the inventory. In particular, the correlations observed for the Personal and Transcendent dimensions suggest that these dimensions of the inventory are quite stable over time. The findings of the test–retest analysis are presented in Table 5. In addition to the test–retest reliability, Cronbach’s alpha values were calculated. Cronbach’s alpha values of Personal, Value, Mystical, Belief, and Transcendence dimensions were found to be 0.83, 0.76, 0.69, 0.55, and 0.24 respectively. The overall inventory exhibited a Cronbach’s alpha of 0.90.

**Table 5:** The dimensions of sprituality inventory—Turkish form test–retest reliability

Scale	Application	X̄	SD	t	sd	p	*r*
Value	Pretest Posttest	54.75 54.77	6.00 6.46	− .026	48	.000	.62**
Belief	Pretest Posttest	23.71 23.91	3.60 3.58	− .350	48	.012	.35*
Mystical	Pretest Posttest	40.51 40.79	4.62 5.23	− .483	48	.000	.65**
Personal	Pretest Posttest	59.22 57.71	6.50 6.01	2.102	48	.000	.68**
Transcendent	Pretest Posttest	22.95 22.87	3.22 3.73	.243	48	.000	.78**
DSI Total	Pretest Posttest	201.16 200.08	18.19 20.30	.547	48	.000	.74**

*p < .05, **p < .001

### Discussion

This study aimed to adapt the Dimensions of Spirituality Inventory (DSI) scale, developed by Wildman et al. (2024), to Turkish to measure spirituality through five subdimensions and 50 questions. The translation process involved 30 experts in English and Turkish, followed by back-translation process. The final Turkish version underwent validity and reliability testing. CFA confirmed the five dimensions of the original scale, Cronbach’s alpha was calculated as 0.90, suggesting high reliability. Only the reliability of the Transcendence dimension was found to be low (α = 0.24). In the analyses conducted to examine the reasons; it was determined that the reliability would increase by removing the Dead Active items in this dimension (α = 0.56). Since the comprehensibility of the items was not found to be problematic in linguistic analysis studies, it was decided to keep the items in the scale in order not to disrupt the integrity of the scale.

The criterion validity study examined the relationship between the DSI and other spirituality scales, such as the Mysticism Scale, Spirituality Scale, Intrinsic Spirituality Scale, and Spiritual Transcendence Scale. Positive correlations supported the theoretical framework, indicating that the Turkish DSI effectively measures the subdimensions of spirituality. Traditional research has focused primarily on religion or religiosity, with spirituality used to describe both mystical and nonreligious practices (Pargament, 2023). These findings affirm that spirituality can be measured multi-dimensionally.

#### Challenges in Measuring Spirituality

One significant challenge in measuring spirituality is determining whether it should be conceptualized as a singular construct or a multidimensional phenomenon. Research indicates that spirituality is not perceived as a universal truth (Ammerman, 2013; Eisenmann et al., 2016; Steensland et al., 2018; Zinnbauer et al., 1997). Various definitions and tools for measuring spirituality exist. For instance, Elkins et al. (1988) concluded that spirituality is a multidimensional structure consisting of nine components. The Spiritual Orientation Inventory (SOI), similar to the DSI, includes subdimensions such as the Transcendent dimension and Meaning and Purpose in Life, which correspond to the Transcendent Beings and Personal subdimensions of the DSI. Many studies that view spirituality as independent of religion and religiosity use the SOI to measure humanistic spirituality, which focuses on individual experiences and personal growth without reference to religious doctrines or institutions (Lazar, 2021).

Nevertheless, scales based on religiosity and religion have also been designed. Given that there are approximately 2 billion Muslims in the world, there has been a concerted effort to create measurement tools that comprehensively represent the affective (attitudes), cognitive (beliefs), and behavioral (practices) aspects of religiosity. One notable example is the Psychological Measure of Islamic Religiousness (PMIR) scale (Abu-Raiya et al., 2008). Unlike many scales designed for specific ethnic or national Muslim populations, the PMIR scale has been employed in research samples across various cross-cultural settings, demonstrating its broader applicability (Amer, 2021). The adaptation and validation of the DSI scale for a Muslim population and its translation into Turkish are significant within the context of the literature, as these efforts enhance the relevance of spirituality measures across different Muslim cultural settings.

#### Spirituality and Mental Health Counseling

The significance of spirituality in addressing mental health challenges has been increasingly recognized. Since most individuals possess some level of religious or spiritual (RS) values, beliefs, or practices that are shaped by their diverse backgrounds, experiences, and identities—and these RS factors can impact their mental health either as a source of strength or challenge—it is essential for mental health professionals to gain a deeper understanding of how clients perceive the integration of RS in their treatment by their providers (Oxhandler et al., 2024).Coppola et al. (2021), during the COVID-19 pandemic, highlighted the connection between spiritual practices and psychological well-being. Research by Karki et al. (2024) explored the relationship between spirituality and mental health across different cultural contexts, including Turkey. Their findings suggest that spirituality and religiosity are linked to reduced symptoms of depression and enhanced life satisfaction. These correlations imply that spiritual beliefs and practices may serve as valuable resources for coping with depressive symptoms and improving overall emotional well-being.

With this adaptation of the DSI scale, it is possible to define and measure spirituality on a national basis in Turkey. This study will support academic and practical studies in psychological counseling, particularly in spirituality. Many scientific studies on spirituality and religiosity have been conducted worldwide, especially in the last 20 years. The role of spirituality in psychological counseling and psychotherapy has gained attention (Shafranske, 2024; Şahin & Okan, 2024). Data from English-speaking Muslims highlight the need to integrate religious and spiritual dimensions into psychological counseling frameworks. According to research conducted by Umarji and Islam (2024), intolerance to uncertainty is identified as the predominant predictor of psychological distress, particularly associated with conditions such as anxiety and depression. In contrast, religiosity is recognized as the most influential predictor of positive psychological outcomes, such as life satisfaction, well-being, and a sense of purpose. Furthermore, religious doubts are found to significantly impact multiple mental health outcomes.

This complexity underlines the necessity of quantifying religiosity to fully comprehend its effects on mental health. With increasing interest in spirituality and mental health, the use of spirituality in the psychological counseling process is beneficial for some clients (Captari et al., 2018; Okan et al., 2024; Post & Wade, 2009). In clients with strong religious and spiritual commitment and engagement in psychological counseling and psychotherapy, therapies focusing on religious and spiritual issues are more effective than those without a religious or spiritual orientation (Bouwhuis-Van Keulen et al., 2024).

Implicitly or explicitly, spiritual issues are often brought into therapy. Spirituality can be experienced in listening to music, the smile of a stranger, the color of the sky, or gratitude for waking up each day. Conversely, spirituality can also be felt in moments of loss, questioning life’s purpose, feeling abandoned, or facing injustice (Pargament, 2011). The items “contemplating beautiful art or music is profoundly spiritually moving and deeply spiritual for me” from the “Appreciating Beauty” subdimension of the DSI are indicative of valid structural features in understanding spirituality (Wildman et al., 2024). However, counselors or therapists may be unaware of other dimensions of spirituality contributing to the therapeutic process, often due to a lack of preparedness to discuss spirituality with clients. Studies indicate that mental health professionals often have incomplete knowledge about the distinctions and commonalities between “religion,” “religiosity,” and “spirituality” (Da Cunha et al., 2024; Okan & Şahin, 2024). This lack of clarity can result in spirituality being neglected or rarely addressed in therapy sessions, impacting session effectiveness, client rapport, and therapist behavior.

Increasing numbers of people in the West identify as “spiritual but not religious,” emphasizing the need to understand cultural and geographical influences on health outcomes related to spirituality. This necessitates considering area-level religious constructs, such as affiliations and community structures, to establish causal relationships between spirituality and health. Additionally, investigating the impact of religiosity and spirituality on psychological health using contemporary scales is crucial (Ransome, 2020). There is a consensus among mental health professionals on the need for training programs to enhance religious/spiritual (R/S) competencies. Vieten et al. (2023) found that most practitioners support the inclusion of competencies to address religious and spiritual struggles and help clients access their R/S strengths. However, one-fifth reported discomfort with these competencies in their professional development. Understanding the subdimensions of spirituality and utilizing the DSI can enhance the effectiveness of psychological counseling sessions. Along with this, effective training in religious and spiritual (RS) integration is essential for mental health professionals to avoid potentially harmful practices and ensure respectful, client-centered care. Common harmful practices include a lack of openness to the client’s RS, imposing their own RS beliefs. Additionally, therapists may exhibit bias against or completely avoid addressing RS, leaving clients feeling disregarded or misunderstood (Oxhandler et al., 2024). Therefore, understanding the subdimensions of spirituality and utilizing the DSI can enhance the effectiveness of psychological counseling sessions. By also increasing spirituality awareness, negative outcomes on both the counselor and client sides can be reduced. This study aimed to assess the suitability of these dimensions within Turkish culture and provide an inventory for mental health professionals to use in their practice.

#### Limitations

There may be a risk of contamination in scales that aim to measure spirituality and religion. As highlighted by Koenig and Carey (2024, 2025) and Bambling (2025), contamination occurs when scales ostensibly measuring religious or spiritual constructs also include items indicative of mental or social health (e.g., meaning, peace, social connectivity). This overlap can lead to tautological associations and artificially inflated correlations, thus reducing the clarity and validity of the findings. In our adapted measure, while the subscale names might initially suggest a contamination risk, item-level analysis indicates they were designed to capture spiritual dimensions directly rather than overall well-being or social connectedness. Nonetheless, researchers should remain mindful of potential contamination and consider the preventative strategies outlined by Koenig and Carey (2025) in future applications.

One of the limitations of this study is the relatively high number of items (53 items in total), which may seem lengthy and time-consuming to participants. An extensive item set may lead to participant fatigue and reduced engagement, affecting the overall quality and generalizability of the data. Future research could consider developing and validating a shorter version of the scale to maintain psychometric rigor while increasing applicability and participant agreement.

Test–retest analyses indicate that the overall scale meets the reliability standard of 0.70 or above, and that most subscales similarly exhibit sufficient test–retest correlations. However, the correlations for the Belief subscale (0.35) and, to some extent, the Value subscale (0.62) falling below 0.70 suggest relatively weaker temporal stability in these dimensions. Additionally, factors such as participants’ recall of previous responses, the relatively short interval between test administrations, and the limited nature of the sample may have influenced the reliability coefficients obtained. Therefore, it is recommended that these findings be replicated over longer time intervals and with different samples to further establish their robustness.

In this study, data collection was designed to minimize biases, but it is challenging to capture all facets of spirituality due to its personal nature. The scale may not thoroughly reflect participants’ perceptions of spirituality. Since this instrument measures various aspects of spirituality, some of the less common specific sub-dimensions (e.g. Dead Active) may not have appealed to the current study group. These dimensions are thought to provide valuable data in some areas of Turkey that this study could not include. CFA was used to validate the subdimensions of spirituality, consistent with the methodologies used by the developers of the scale. However, reapplication of exploratory factor analysis (EFA) and confirmatory factor analysis (CFA) is necessary to address cultural suitability and unexpected structural findings.

#### Future Research

Further research is needed to investigate the effectiveness of individual DSI scale items and to create culturally valid item groupings, especially for samples from Islamic traditions. Identifying cultural equivalents for certain subdimensions presents notable challenges. Future studies should explore the relationship between the Turkish form of the DSI and various positive psychology concepts and psychopathological conditions. The development of practical guidelines for mental health professionals to incorporate spirituality and religion into their practice is recommended. This may lead to research on the benefits of religious or spiritual activities as coping strategies.

#### Conclusion

Our evidence supports the psychometric validity of the DSI-Turkish form in a heterogeneous group of Turkish participants. Future research is recommended to confirm our findings and to identify the extent of spiritual needs in individuals who prioritize spiritual well-being. This study’s findings highlight the necessity for culturally sensitive tools to capture the diverse expressions of spirituality across different cultural settings. This contribution is expected to enhance both academic and practical applications in the field of psychological counseling in Turkey, particularly concerning spirituality.

## Appendix 1

See Table 6.

**Table 6:** Items of the dimensions of spirituality inventory (Wildman et al., 2024)

Item code	Item statement (EN-TR)	Subdimensions	Main dimensions
m1	Usually, when I feel most spiritually alert, I am also intensely aware of my body. *(Manevî olarak aktif olduğum zamanlarda—meditasyon uygulamaları gibi- genellikle bedenimin de yoğun bir şekilde farkında olurum.)*	Kinesthetic/kinaesthetic	Value ideals
m49	My spirituality involves striving to maintain continual awareness of my body while performing my daily activities. *(Maneviyatım, günlük işlerimi yaparken, bedenim üzerine daimî bir farkındalığı koruma çabasını içerir.)*	Kinesthetic/kinaesthetic	
m16	Contemplating beautiful art or music is profoundly spiritually moving for me. (*Benim için sanattaki veya müzikteki güzelliği derinlemesine deneyimlemek son derece manevîdir.)*	Appreciating beauty	
m48	Intense experiences of beauty in art or music are deeply spiritual for me. (*Güzel bir sanat eseri ya da müzik üzerine tefekkür etmek/düşünmek beni manevî anlamda derinden etkiler.)*	Appreciating beauty	
m14	My spirituality draws me toward beautiful art and music. (Maneviyatım beni güzel olan sanat eserlerine ve müziğe çeker.)	Appreciating beauty	
m34	When I am not spiritually attuned, I fail to appreciate the value of the people and things around me. (*Manevî insicam/uyum yakalamadığımda, etrafımdaki insanlar ve diğer şeylerin kıymetini takdir etmekte başarısız olurum.)*	Axiological sensitivity	
m29	My appreciation for people and things around me rises and falls with my spiritual vitality. *(Etrafımdaki insanların ve diğer şeylerin kıymetine olan takdirim, manevi şevkimle/ canlılığımla birlikte artar veya azalır.)*	Axiological sensitivity	
m13	My spirituality increases my gratitude for the people and things in my life. *(Maneviyatım, hayatımdaki insanlara ve diğer şeylere duyduğum şükranı artırır.)*	Axiological sensitivity	
m35	When I am spiritually attuned, I find it easier and more natural to be an ethical person. *(Manevî insicam/uyum yakaladığımda, etik bir insan olmak daha kolay ve doğal gelir.)*	Ethical growth	
m41	Due to spiritual insights, I have adopted ethical standards for myself that are higher than those I previously adhered to. *(Manevî içgörü/farkındalıklar sayesinde, kendim için daha önce bağlı olduğumdan daha yüksek ahlaki ilkeler benimsemiş oldum.)*	Ethical growth	
m51	My spirituality makes me a better person. *(Maneviyatım beni daha iyi bir insan yapıyor.)*	Ethical growth	
m17	My spirituality increases my desire for intellectual understanding. *(Maneviyatım, entelektüel kavrayışa olan arzumu arttırır.)*	Truth seeking	
m33	Pursuing intellectual understanding is a central part of my spirituality. *(Entelektüel kavrayışın peşinde olmak maneviyatımın merkezi bir parçasıdır.)*	Truth seeking	
m44	Pursuing intellectual understanding is not central to my spirituality. *(Entelektüel kavrayışın peşinde olmak maneviyatımın merkezi bir parçası değildir.)*	Truth seeking	
m32	Most of my deepest personal relationships have been formed in spiritual communities.* (En samimi kişisel ilişkilerimin çoğu, manevî topluluklarda kurulmuştur.)*	Connection	Personal
m42	Connection to my spiritual community is essential to my spiritual fulfillment.* (Manevi topluluğuma olan aidiyetim, manevî doyumumda esastır.)*	Connection	
m38	Connection to my spiritual community is not essential to my spiritual fulfillment.* (Manevi topluluğuma olan aidiyetim manevî doyumumda esas değildir.)*	Connection	
m7	My spirituality provides the deepest meaning and purpose in my life.* (Maneviyatım hayatımdaki en yüce anlamı ve amacı sağlar.)*	Meaning	
m36	My spirituality gives my life a clear sense of meaning and direction.* (Maneviyatım hayatıma açık/net bir anlam ve istikamet katar.)*	Meaning	
m43	My spirituality helps me to accept change, even when change is painful. *(Maneviyatım, acı verici olsa bile değişimi kabul etmeme yardımcı olur.)*	Nonattachment	
m27	Accepting change, even when it is difficult, is vital to my spirituality. *(Zor olsa bile değişimi kabul etmek, maneviyatım için hayatî önem taşır.)*	Nonattachment	
m37	Accepting change, even when it is difficult, is not central to my spirituality. *(Zor olsa bile değişimi kabul etmek, maneviyatımın merkezinde yer almaz.)*	Nonattachment	
m6	Making habits out of spiritual disciplines such as meditation, prayer, study, or self-reflection is the key to my spiritual development.* (Meditasyon, dua veya kendi üzerine düşünme / nefis muhasebesi gibi manevi disiplinlerden alışkanlıklar edinmek manevi gelişimimin anahtarıdır.)*	Practices	
m40	Personal practices such as meditation, prayer, study, or self-reflection are absolutely necessary for my spiritual life. *(Meditasyon, dua, kendi üzerine düşünme /nefis muhasebesi gibi kişisel uygulamalar, manevî hayatım için gereklidir.)*	Practices	
m10	Finding my true self is the key to spiritual fulfillment.* (Gerçek benliğimi bulmak, manevî doyuma ulaşmamın anahtarıdır.)*	Self-discovery	
m12	My spirituality focuses on discovering my truest, deepest self.* (Maneviyatım, benliğimin en hakikî, en derin hâlini keşfetmeyi hedefler.)*	Self-discovery	
m45	My spirituality helps change my behavior for the better. *(Maneviyatım, davranışlarımı daha iyi yönde değiştirmeme yardım eder.)*	Self-transformation	
m20	Due to my spirituality, I have been transformed into a better person. *(Maneviyatım sayesinde daha iyi bir insana dönüştüm.)*	Self-transformation	
m39	My life would be much harder without spiritual beliefs that guide me.* (Bana rehberlik eden manevî inançlarım olmasaydı hayatım çok daha zor olurdu.)*	Belief	Belief and belonging
m53	I need core spiritual beliefs if my life is to have structure and direction.* (Hayatımda düzen ve istikamet için temel manevi inançlara ihtiyaç duyarım.)*	Belief	
m50	Traditional rituals enhance my spiritual life in profound and powerful ways.* (Geleneksel ritüeller/âdetler manevi yaşamımı derin ve güçlü bir şekilde artırır.)*	Ritual	
m46	Traditional rituals hinder my spiritual life more than they help it.* (Geleneksel ritüeller/âdetler manevî hayatımı desteklemekten ziyade engeller.)*	Ritual	
m47	My religious tradition guides my spiritual journey*. (Dinî geleneğim manevî serüvenime rehberlik eder.)*	Religous tradition	
m23	My spirituality and my religious tradition are intertwined.* (Maneviyatım ve dinî geleneğim iç içe geçmiştir.)*	Religous tradition	
m31	Awe before the overwhelming immensity of cosmic reality is spiritually vital for me. (Kâinatın insanı hayrete düşüren sonsuzluğu karşısında huşu duymak benim için manevî açıdan hayatî önem taşır.)	Awe	Mysticism
m3	Hayatımı, kâinatın sonsuz okyanusunda tek bir su damlası olarak düşünmek beni hayret duygusuyla doldurur. (Picturing my life as (continued) a single drop of water in the endless ocean of cosmic reality fills me with a sense of wonder.)	Awe	
m8	Contemplating the awesome vastness of the cosmos is spiritually energizing to me. (Evrenin muazzam enginliğini tefekkür etmek/düşünmek manevi şevkimi artırır.)	Awe	
m4	The deepest spiritual questions are too mysterious for human beings to answer*. (En derin manevî sorular insanlığın yanıtlayamayacağı kadar gizemlidir.)*	Mystery	
m11	No human being can answer all of the spiritual questions that matter to us.* (Hiçbir insan, bizim için önem taşıyan manevî soruların tümüne cevap veremez.)*	Mystery	
m9	My spirituality increases my sense that reality is fundamentally mysterious. *(Maneviyatım, gerçekliğin özünde gizemli olduğuna dair hissiyatımı artırır.)*	Mystery	
m30	My spirituality involves a growing sense of being one with all things.* (Maneviyatım, her şeyle bir olma duygusunun giderek artmasını içerir.)*	Oneness-unity	
m18	My spirituality gives me a feeling of unity with all of reality.* (Maneviyatım bana bütün gerçeklikle bir olma duygusu verir.)*	Oneness-unity	
m19	My spirituality involves potent experiences of transcendence.* (Maneviyatım güçlü aşkınlık deneyimleri içerir.)*	Oneness-transcendence	
m25	My spirituality does not involve potent experiences of transcendence.* (Maneviyatım güçlü aşkınlık deneyimleri içermez.)*	Oneness-transcendence	
m15	I believe we are immersed in a realm of unseen spiritual powers, both good and bad.* (Gözle görünmeyen hem iyi hem de kötü ruhanî güçlerin olduğu bir âlemle iç içe olduğumuza inanıyorum.)*	Spiritual beings	Transcendent beings
m28	In order to make sense of the world, I need to recognize the power of both good and evil spirits.* (Dünyayı anlamlandırabilmek için hem iyi hem de kötü ruhların gücünü kabul etmem gerekir.)*	Spiritual beings	
m52	My spirituality is perfectly meaningful without any Divine being.* (Herhangi bir ilahî varlık olmadan da maneviyatım son derece anlamlıdır.)*	Divine beings	
m26	My spiritual experiences tend to involve sensing the presence of the Divine.* (Manevî deneyimlerimin çoğunda İlahî olanın huzurunda bulunduğumu hissederim.)*	Divine beings	
m24	I believe the dead are not gone but directly influence the living.* (Ölülerin bu dünyadan gitmediğine ve yaşayanlara doğrudan etki edebildiklerine inanıyorum.)*	Dead active	
m22	I believe the dead are gone and have no direct influence on the living.* (Ölülerin bu dünyadan gittiğine ve yaşayanlara doğrudan bir etkilerinin olmadığına inanıyorum.)*	Dead active	
m2	Under ordinary circumstances, and all things being equal, rich people should be considered spiritually superior to poor people.* (Diğer tüm şartların aynı olduğu varsayılırsa, zengin insanlar fakir insanlardan manevî anlamda üstün sayılmalıdır.)*	Control and calibration items	
m5	Under ordinary circumstances, and all things being equal, it is better to be kind than cruel.* (Diğer tüm şartların aynı olduğu varsayılırsa, şefkatli olmak gaddar olmaktan daha iyidir.)*		
m21	Under ordinary circumstances, and all things being equal, physically attractive people should be considered spiritually superior to unattractive people.* (Diğer tüm şartların aynı olduğu varsayılırsa, bedenen güzel insanların, güzel olmayan insanlardan manevî olarak üstün olduğu düşünülmelidir.)*		
